# Odd-skipped labels a group of distinct neurons associated with the mushroom body and optic lobe in the adult *Drosophila* brain

**DOI:** 10.1002/cne.23375

**Published:** 2013-09-25

**Authors:** Peter Levy, Camilla Larsen

**Affiliations:** Medical Research Council Centre for Developmental Neurobiology, King's College LondonLondon, United Kingdom

**Keywords:** *Drosophila*, CNS, visual system, inferior protocerebrum, mushroom body

## Abstract

Olfactory processing has been intensively studied in *Drosophila melanogaster*. However, we still know little about the descending neural pathways from the higher order processing centers and how these connect with other neural circuits. Here we describe, in detail, the adult projections patterns that arise from a cluster of 78 neurons, defined by the expression of the Odd-skipped transcription factor. We term these neurons Odd neurons. By using expression of genetically encoded axonal and dendritic markers, we show that a subset of the Odd neurons projects dendrites into the calyx of the mushroom body (MB) and axons into the inferior protocerebrum. We exclude the possibility that the Odd neurons are part of the well-known Kenyon cells whose projections form the MB and conclude that the Odd neurons belong to a previously not described class of extrinsic MB neurons. In addition, three of the Odd neurons project into the lobula plate of the optic lobe, and two of these cells extend axons ipsi- and contralaterally in the brain. Anatomically, these cells do not resemble any previously described lobula plate tangential cells (LPTCs) in *Drosophila*. We show that the Odd neurons are predominantly cholinergic but also include a small number of γ-aminobutyric acid (GABA)ergic neurons. Finally, we provide evidence that the Odd neurons are a hemilineage, suggesting they are born from a defined set of neuroblasts. Our anatomical analysis hints at the possibility that subgroups of Odd neurons could be involved in olfactory and visual processing.

A central objective in neuroscience is to understand the neural mechanisms that translate a sensory stimulus into a behavioral response. Crucial to understanding sensory processing is an anatomical description of the essential circuit components—the various cell types and how they connect to one another. This is inherently difficult in the *Drosophila melanogaster* brain as there are few morphological landmarks by which to identify specific neural groups. However, the advent of genetic techniques such as the Gal4/UAS system and other binary systems (Brand and Perrimon, 1993; Potter et al., 2010) has enabled us to faithfully label and manipulate defined neuronal populations within the central nervous system (CNS). In fact, the necessity for generating such tools has led to the systematic generation of fly lines in which all neurons in the brain can be uniquely labeled (Pfeiffer et al., 2008, 2010). Here we exploit similar strategies in *Drosophila* to identify novel cellular components that may be involved in olfactory processing.

In the olfactory system volatile odors are recognized by olfactory receptor neurons (ORNs), which project to the antennal lobe (AL) where they synapse on to projection neurons (PNs), thereby forming several glomeruli (Stocker, 1994; Wilson et al., 2004). From the AL PNs carry odor information to the higher order processing centers, the mushroom bodies (MB) and lateral horn (LH). The MBs are composed of a group of neurons termed Kenyon cells that project dendrites into the calyx, whereas the axons form several horizontal and vertical lobes within the superior and inferior protocerebrum. The MB is required for olfactory learning and memory (Davis, 1993; Heisenberg et al., 1985; Turner et al., 2008), but how reception of odor information in the MB is translated into behavior output is largely unknown (Davis, 1993; Heisenberg et al., 1985; Turner et al., 2008). However, recently a number of extrinsic MB neurons have been implicated in different aspects of learning and memory (Liu et al., 2012; Pitman et al., 2011; Sejourne et al., 2011). Common to most extrinsic MB neurons is that they receive input from the Kenyon cell axons and send axons to other parts of the brain (Ito et al., 1998).

Information from other sensory modalities can influence olfactory output. For example, it has been shown both in free flight and tethered flight experiments that *Drosophila* requires visual and olfactory integration to track an odor plume effectively (Chow et al., 2011; Frye et al., 2003). In flying insects visual motion input is sampled by a group of neurons collectively termed lobula plate tangential cells (LPTCs) (Borst et al., 2010). In *Drosophila* the only LPTCs so far identified are the HS and VS cells that respond to wide-field motion either along the horizontal or along the vertical axis.

We have previously described a group of neurons in the larvae that are characterized by the expression of the Odd-skipped transcription factor. Interestingly, these neurons project into the calyx of the MB in the larvae (Larsen et al., 2006), suggesting a role in olfactory learning and memory. In this study we refer to these cells as Odd neurons. Here we extend the descriptive study of the Odd neurons by examining their projection pattern in the adult brain. In particular we are interested to know whether the projections into the olfactory system are maintained in the mature CNS. Such a connection would imply that the Odd neurons could play a role in olfactory processing in the adult.

We find that in the adult brain there are 78 Odd neurons, clustered together in the posterior–lateral part of the brain. By using genetically encoded markers, we have mapped the dendritic and axonal projection patterns and show that some of the Odd neurons project dendrites into the calyx and axons into the inferior protocerebrum (IPR), ventromedial protocerebrum (VMPR), and ventrolateral protocerebrum (VLPR). We also identify the different neurotransmitters expressed in the Odd neurons. Using the MARCM (Mosaic analysis with a repressible cell marker) (Lee and Luo, 2001) approach, we show that the Odd cell cluster contains three groups of neurons. In addition to the group that projects into the calyx of the MB, one group projects into the lobula plate of the optic lobe and another group projects exclusively within the IPR, VLPR, VMPR, and PLPR. We show that some of the Odd neurons that project into the MB are likely a previously uncharacterized group of extrinsic MB neurons. Likewise, the neurons that project into the optic lobe belong to a novel group of lobula plate tangential cells (LPTCs). Finally, we use a combination of MARCM clones and neuroblast (Nb) markers to address how the Odd lineage is generated.

## MATERIALS AND METHODS

### Fly strains and genetics

All *Drosophila melanogaster* fly strains reported here were kept at 25°C on standard fly food. Characterization of the Odd neural projection pattern was performed by using the *Odd-Gal4* (CL) line (Larsen et al., 2006). Identification of axons and dendrites was achieved by using *Odd-Gal4* (kind gift of Fernando Casares) (Bras-Pereira et al., 2006) to drive UAS-*synaptotagmin-GFP* (Zhang et al., 2002) (Bloomington Stock Center, Bloomington, IN), UAS-*DenMark* (kind gift of Hassan Bassem) (Nicolai et al., 2010), and UAS-*Bruchpilot* (Smith and Taylor, 2011). We found that the cross between *Odd-gal4* and UAS-*DenMark* was lethal at larval stages and therefore used a *Tub-Gal80ts* (Bloomington Stock Center) to suppress DenMark until adult stages. To address whether Kenyon cells express *Odd-skipped*, we used a line containing a LacZ insertion in the Odd-skipped locus: Odd^rk111^ (kind gift of Cordelia Rauskolb) (Hao et al., 2003) and the Kenyon driver *OK107-Gal4* (Bloomington Stock Center) and UAS-*CD8GFP* (Bloomington Stock Center). MARCM clones (Lee and Luo, 2001) were generated by using the following stocks: *FRT19A* (Bloomington Stock Center) *Odd-Gal4*, UAS-*CD8GFP* and *FRT19A,hsFLP,Tub-Gal80* (Bloomington Stock Center). Expression of neurotransmitters was assessed by using the *Chat-Gal4* line (Salvaterra and Kitamoto, 2001) (Bloomington Stock Center) and *DVGlut-Gal4* (Daniels et al., 2008) (Bloomington Stock Center). Colocalization between the Odd neurons and PN neurons was addressed by using the *GH146-QF.P* line (Bloomington Stock Center) crossed to *QUAS-mtdTomato-3xHA* (Bloomington Stock Center) (Potter et al., 2010) in combination with the Odd-Gal4 (CL) line. Colocalization between the Odd neurons and the HS/VS cells was addressed by using the 3A-Gal4 line (Scott et al., 2002) together with the Odd^rk111^ line.

### Antibodies

See Table1 for a list of antibodies.

**Table 1:** Primary Antibodies Used in This Study

Name	Immunogen	Commercial supplier	Dilution
Nc82	*E. coli*-derived recombinant *Drosophila* Bruchpilot	Monoclonal mouse anti-bruchpilot. Developmental Studies Hybridoma Bank, Iowa City, IA, Cat. No. Nc82	1:10
β-Galactosidase	Full-length native protein (purified)	Polyclonal chick anti-β-Gal Abcam, Cambridge, MA, Cat. No. ab9361	1:2,000
1D4	*E. coli*-derived recombinant *Drosophila* Fasciclin II containing terminal 496 amino acids	Monoclonal mouse anti-fasciclin II. Developmental Studies Hybridoma Bank, Cat. No. 1D4	1:10
C458.2H	*E. coli*-derived recombinant *Drosophila* Notch extracellular domain repeats 12–20	Monoclonal mouse anti-notch. Developmental Studies Hybridoma Bank, ICat. No. C458.2H.	1:10
GFP (rabbit)	Full-length native protein (purified)	Polyclonal rabbit anti-GFP. Invitrogen, La Jolla, CA, Cat. No. 11122.	1:500
GFP (mouse)	*E. coli*-derived recombinant full-length GFP	Monoclonal mouse anti-GFP. Roche, Indianapolis, IN, Cat. No. 1814460	1:500
GABA	γ-Aminobutyric acid (GABA) conjugated to BSA	Polyclonal rabbit anti-GABA. Sigma, St Louis, MO, Cat. No. A2052	1:100
MR1A	GST fusion protein containing amino acid residues 1,196–1,320 of prospero	Monoclonal mouse anti-prospero. Developmental Studies Hybridoma Bank, Cat. No. MR1A	1:10
Deadpan	GST fusion protein containing amino acid residues 109–365 of deadpan	Polyclonal rabbit anti-deadpan (gift from James Skeath)	1:500
8D12	Full-length protein	Monoclonal mouse anti-reversed polarity. Developmental Studies Hybridoma Bank, Cat. No. 8D12	1:10
ChAT4B1	Recombinant full-length ChAT fusion protein	Monoclonal mouse anti-choline acetyltransferase, Developmental Studies Hybridoma Bank, Cat. No. ChAT4B1	1:10
dsRed	Full-length dsRed-express protein	Polyclonal rabbit anti-dsRed, ClonTech, Mountain View, CA, Cat. No. 632496	1:500
Odd-skipped	Full-length protein	Polyclonal guinea-pig anti-odd-skipped, Asian Center for Segmentation Antibodies	1:100
DVGLUT	Peptide encoding amino acids 620–632 of DVGLUT	Polyclonal rabbit anti-vesicular glutamate transporter (kind gift from Aaron Diantonio)	1:1,000

#### NC82

The nc82 antibody is made by the Developmental Studies Hybridoma Bank (DSHB; Iowa City, IA; nc82, donated by E. Buchner). It is a mouse monoclonal antibody and recognizes the protein Bruchpilot (Wagh et al., 2006). The antibody was raised using *Drosophila* head homogenate and has been characterized using western blots where the antibody specifically recognizes 190- and 170-kDa bands. These bands disappear in Bruchpilot knock-down flies (Wagh et al., 2006). We used the supernatant at a dilution of 1:10.

#### Anti-β-galactosidase

This β-galactosidase antibody is manufactured by AbCam (Cambridge, MA; Cat. No. ab9361) and is a polyclonal chicken antibody raised against the full-length native *Escherichia coli* protein and immunoaffinity purified (AbCam technical information). The antibody has been used previously against transgenically expressed β-galactosidase in *Drosophila* (Tuxworth et al., 2009) and does not label nonexpressing cells. We used a 1:2,000 dilution.

#### Anti-FasII

The anti-FasII antibody is a mouse monoclonal antibody made by DSHB (1D4, donated by C. Goodman). The antibody was raised against a fusion protein containing the C-terminal 496 amino acids, which includes the cytoplasmic domain. The staining disappears in FasII knock-out flies (Grenningloh et al., 1991). We used the supernatant at a dilution of 1:10.

#### Anti-Notch

Anti-Notch (DSHB #C458.2H, donated by S. Artavanis-Tsakonas) is a mouse monoclonal antibody generated against the extracellular domain of the *Drosophila* Notch protein. The antibody was raised against a fusion protein including the EGF repeats 12–20 of the Notch protein. Specificity of the antibody was assessed by western blot comparing Notch transfected S2 cells and nontransfected S2 cells. The antibody specifically recognizes a predicted band size of 300 kDa. This band is absent in nontransfected cells (Fehon et al., 1990; Okajima and Irvine, 2002). We used the supernatant at a dilution of 1:10.

#### Anti-GFP (rabbit)

The anti-GFP antibody (Invitrogen, La Jolla, CA, #11122) is a rabbit polyclonal raised directly against green fluorescent protein (GFP) isolated from *Aequorea victoria*. It was purified by ion-exchange and has been used previously in *Drosophila* to label genetically expressed GFP by comparing localization of expression in specimens with and without GFP expression (Kamikouchi et al., 2006). We used this antibody at a 1:500 dilution.

#### Anti-GFP (mouse)

Mouse anti-GFP is a mixture of two high-affinity monoclonal antibodies (Roche, Indianapolis, IN, #1814460). They were raised against partially purified recombinant GFP. They detect specifically GFP and GFP-fusion proteins (manufacturer's notes). This antibody has been used previously in *Drosophila* to detect genetically expressed GFP by comparing localization of expression in specimens with and without GFP expression (Miyazaki and Ito, 2010). We used a 1:500 dilution.

#### Anti-GABA

This antibody is a rabbit polyclonal and is manufactured by Sigma (St. Louis, MO, #A2052). It was raised against a conjugate of γ-aminobutyric acid (GABA)–bovine serum albumin and affinity immunopurified. The antiserum was characterized by dot-blot immunoassays by the manufacturer and has been used to label GABA protein in *Drosophila* brains previously (Wilson et al., 2004). We used this antibody at a 1:100 dilution

#### Anti-Prospero

The antibody against Prospero is available from DSHB (#MR1A, donated by C.Q. Doe). The antibody is mouse monoclonal raised against the prospero amino acids sequence 1,196–1,320 as a GST fusion protein; a hybridoma line was generated. Immunolocalization of this antibody is similar to that described for both *prospero* mRNA localization and other anti-prospero antibodies documented in the literature. (Spana and Doe, 1995). We used the supernatant at a dilution of 1:10.

#### Anti-Deadpan

The anti-Deadpan is a rabbit polyclonal and was a kind gift from James Skeath. It is raised against a fusion protein containing amino acids 109–365 of the deadpan protein. The isolated fusion protein was used as an immunogenic and the antibody was purified with affinity purification by using deadpan fusion protein. Specificity of the antibody was confirmed by comparing expression with that of a LacZ insertion in the deadpan gene. This antibody has been used by several labs to specifically label Deadpan-expressing cells in *Drosophila* (Bier et al., 1992). The antibody was used at a dilution of 1:500.

#### Anti-Repo

Anti-Repo is a mouse monoclonal antibody from DSHB (#8D12, donated by C. Goodman) that specifically recognizes the *Drosophila* protein reversed polarity (Repo). It was generated against bacterially expressed full-length protein. Specificity of the antibody was tested by comparing antibody staining between wild-type flies and repo-null mutant flies. This antibody is routinely used in *Drosophila* to label Repo-expressing cells. (Spokony and Restifo, 2009; Xiong et al., 1994). We used the supernatant at a 1:10 dilution.

#### Anti-choline acetyltransferase

This antibody recognizes specifically *Drosophila* choline acetyltransferase and is used as a marker for cholinergic neurons. It is a mouse monoclonal antibody produced by DSHB (#ChAT4B1, donated by A. Salvaterra). Antibody specificity was confirmed by western blotting where the antibody recognizes a 83-kDa band that is the predicted size of the fusion protein used to generate the antibody (Takagawa and Salvaterra, 1996). We used the supernatant at a 1:10 dilution.

#### Anti-DVGLUT (glutamate)

The DVGLUT antibody recognizes *Drosophila* vesicular glutamate transporter and is used to label glutamatergic neurons. This antibody was a kind gift of Dr Aaron Diantonio. It is a rabbit polyclonal antibody raised against a peptide encoding amino acids 620–632 of DVGLUT and affinity purified. Antibody specificity was confirmed by reduced staining on tissue and western blot from a hypomorphic allele of DVGLUT (Daniels et al., 2008). We used the antibody at a dilution of 1:1000

#### Anti-dsRed

This rabbit polyclonal antibody from ClonTech (Mountain View, CA, #632496) targets *Discosoma* red fluorescent protein (RFP). In flies where RFP is targeted to epithelial glia using *pfFRC71*antibody binding is seen in the known expression pattern of *pfFRC7*. In addition, antibody labeling is absent in flies in which RFP is not expressed (Edwards et al., 2012). We used this antibody at a 1:500 dilution.

#### Anti-Odd-skipped

The odd-skipped antibody was raised in guinea pig using the full-length protein as an antigen. It was obtained through the Asian distribution center for segmentation antibodies and Professor Herbert Jackle. The specificity of the serum was confirmed by staining of *Drosophila* embryos, which resulted in a similar pattern of Odd-skipped expression as that reported previously in the literature. Furthermore, staining is absent in Odd-skipped null mutant embryos (Kosman et al., 1998). We used the serum at a 1:100 dilution.

### Secondary antibodies

Secondary antibodies (Invitrogen, La Jolla, CA) were: Alexa Fluor 488 donkey anti-rabbit, Alexa Fluor 488 goat anti-mouse, Alexa Fluor 546 donkey anti-chick, Alexa Fluor 546 goat anti-mouse, Alexa Fluor 546 goat-anti-rabbit, and Alexa Fluor 633 donkey anti-rabbit. Secondary antibodies were used at a 1:500 dilution.

### Immunohistochemistry

Adult, pupal, and larval brains were dissected in cold phosphate-buffered saline (PBS; pH 7.2) and fixed in PBS-buffered 4% formaldehyde for 30 minutes. Brains were washed several times in PBS and stored in methanol at 20°C. Standard antibody labeling was followed (Ashburner, 1989). Briefly, brains were rehydrated in PBS containing 0.5% Triton X-100 (Sigma; PBS/Triton) and incubated in PBS/Triton plus 10% goat serum (Sigma) for 3 hours. Brains were incubated with primary antibody for 2 days and secondary antibody overnight. The brains were washed several times in PBS/Triton between the primary and secondary antibody. A similar protocol was used for the DV-Glut antibody except brains were fixed in Boyen's solution followed by several washes in PBS before incubation with goat serum. Images were captured by using a Zeiss 510 confocal microscope.

### Embryo collection and Immunohistochemistry

Flies were allowed to lay eggs on yeasted apple juice agar plates for 12 hours. Embryos were collected, dechorinated in bleach, and fixed in PBS-buffered 4% formaldehyde. Antibody staining was performed as for adult brains except Tween-20 (Sigma) was used instead of Triton X-100. Also primary antibodies were incubated overnight and secondary antibodies for 2 hours.

### MARCM clones

MARCM clones were generated as previously described (Lee and Luo, 2001). Briefly, eggs were collected for 1 hour on yeasted apple juice agar plates and allowed to develop at 25°C. Heat shock was applied at different stages of development for 45 minutes at 37°C. Heat-shocked embryos and larvae were allowed to develop for 2 days at 25°C. Larvae were then screened for GFP expression and positive larvae were transferred to vials containing standard fly food and allowed to develop at 25°C until the desired developmental stage. Brains were dissected in a similar manner as for immunohistochemistry.

### Retrograde DiI labeling

The neurons that project to the antennal mechanosensory and motor center (AMMC) originate predominantly from the Johnston organ situated at the second antennal segment. To specifically label these neurons we used DiI (1,1′-dioctadecyl-3,3,3′,3′-tetramethylindocarbocyanine perchlorate; Invitrogen) at a working dilution of 6 μg/μl in DMF (dimethylformamide). Adult Odd-gal4 UAS *CD8-GFP* flies were anesthetized with CO_2_ and the third antennal segment was removed. The second antennal segment was crushed and DiI was applied to the crushed tissue by using a fine needle. Flies were allowed to recover and maintained at 25°C for 24 hours. Brains were then dissected in cold PBS and directly viewed by using a Zeiss 510 confocal microscope.

### Image acquisition

All images were taken by using a Zeiss 510 confocal microscope. Unless otherwise stated, all images were acquired as a z-stack with 2-μm intervals between each confocal section. Images were acquired by using either 20× air or 40× oil immersion objectives at a resolution of 512 × 512 pixels. The degree of further magnification is given in the figure legends. Image J was initially used to generate z-stacks or individual sections of the images acquired. Beyond this there was little image manipulation apart from general adjustment of brightness and contrast in Photoshop (Adobe Systems, San Jose, CA).

## RESULTS

### Odd-skipped driver lines

**Figure 1:**
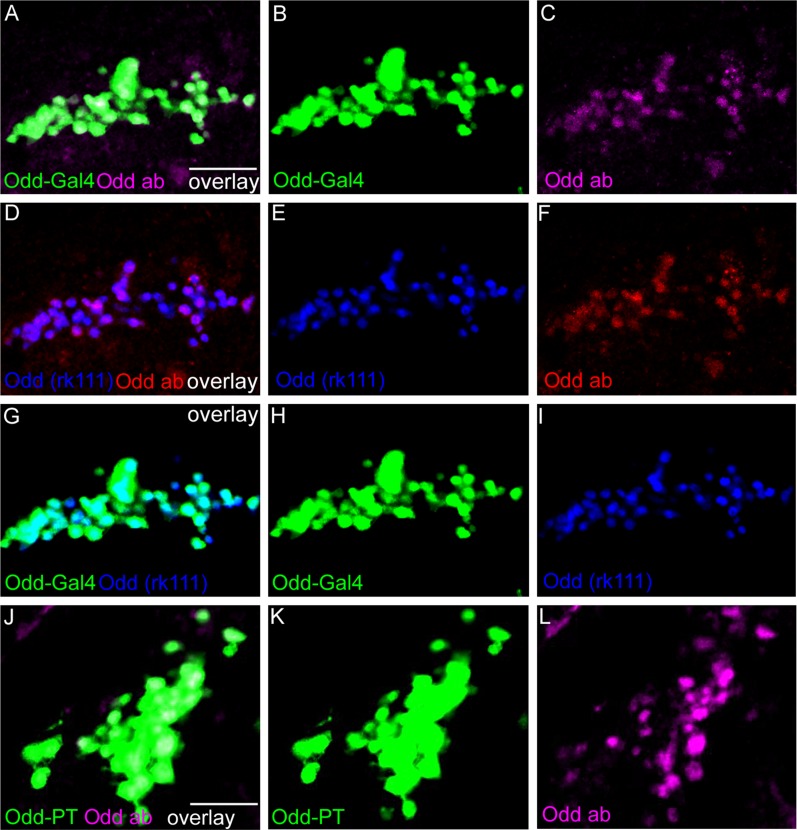
Colocalization among Odd-skipped Gal4 drivers, Odd^rk111^, and Odd-skipped protein. All images are posterior views of adult brains, with lateral to the left and dorsal up. They are ×40 magnification with a further zoom of 1.5. A: Colocalization between the Odd-Gal4 driver (green) and an antibody against Odd-skipped (red). Each image is a maximum intensity projection composed of six confocal sections (total depth of 12 μm). The expression pattern of the Odd-Gal4 line overlaps completely with the antibody against Odd-skipped. B,C: Single fluoroform. D: Colocalization between Odd^rk111^ line (blue) and an antibody against Odd-skipped (red). E,F: Single fluoroform. The expression pattern of the fly line also overlaps with the antibody. G: Colocalization between the Odd^rk111^ (blue) and the Odd-Gal4 line (green). H,I: Single fluoroform. Both fly lines are expressed in the same cells. J: Colocalization between Odd-PT line (green) and the antibody against Odd-skipped (magenta). Each image is a maximum intensity projection composed of eight confocal sections, withe a total depth of 16 μm. Odd-skipped antibody localization (magenta) is seen in all the Odd-PT expressing cells (green). K,L: Single fluoroform. Scale bar = 50 μm in A (applies to a–I) and J (applies to J–L). [Color figure can be viewed in the online issue, which is available at wileyonlinelibrary.com.]

For this study we used a number of genetic drivers expressed specifically in the Odd-skipped–expressing neurons. To ensure that these driver lines faithfully recapitulate Odd-skipped expression, we compared the expression of the driver lines with that of an antibody specific to Odd-skipped (Kosman et al., 1998). In this study we label Odd neurons by using a previously described Gal4 insertion in which GFP is targeted to the membrane of the Odd cells (Odd-Gal4 (cl)), thus allowing a detailed dissection of neuronal arbor morphology (Larsen et al., 2006). To address colocalization between the Odd neurons and Gal4 drivers that are specific for different neurotransmitters, we have taken advantage of the Odd^rk111^ line, which is an insertion of the LacZ gene in the Odd locus (Hao et al., 2003). In this line, LacZ is driven by the Odd-skipped enhancer and is therefore expressed in a similar pattern to Odd-skipped. This allows us to use Gal4 driver lines while independently labeling Odd neurons. The Odd^rk111^ line was also used to address whether the Odd neurons are part of the Kenyon cell cluster as well as the HS/VS LPTCs. Finally, we also used an Odd-Gal4 line that does not contain endogenous GFP expression (Bras-Pereira et al., 2006). This line was used when coexpressing UAS-DenMark and Synaptotagmin-GFP, as endogenous GFP would have masked the localization of Synaptotagmin. This Odd-Gal4 line was also used to generate MARCM clones, as the Odd-Gal4 (cl) line contains endogenous FRT sites. The expression of these three driver lines overlap precisely with the expression of the Odd-skipped protein as assessed by antibody staining (Fig. 1A–F, J–L). Furthermore, the expression of two of the Odd driver lines (Odd^rk111^ line and Odd-Gal4) also overlaps (Fig. 1G–I). This further confirms that the neurons labeled by these three driver lines all express Odd-skipped protein. The expression of the Odd-Gal4 (cl) line overlaps precisely with the Odd-skipped specific antibody (Fig. 1J–L). We are therefore confident that these three fly lines faithfully recapitulate endogenous Odd-skipped expression.

### The Odd cells are predominantly cholinergic neurons that project into specific brain compartments

**Figure 2:**
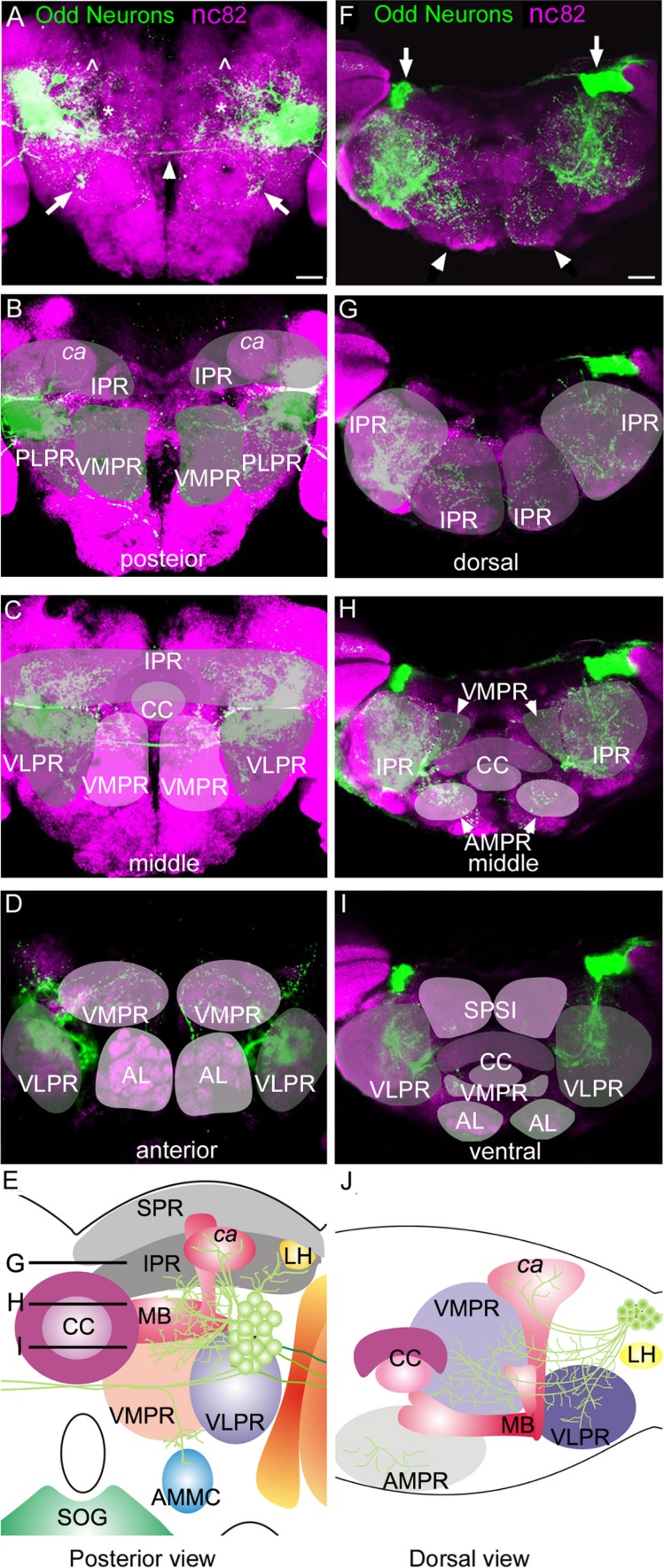
Projection pattern of the Odd neurons. All images are ×20 magnification. A,F: Confocal maximum intensity projection images composed of (A) 20 sections (total depth of stack 40 μm) and (B) 35 sections (total depth of stack 70 μm). Images shown in B–D and G–I are each composed of two confocal sections (4 μm thickness) at different (B–D) anterior–posterior and (G–I) dorsoventral levels. The position of each dorsoventral section is shown in E. In all confocal images Odd neurons are labeled in green, and the neuropil is visualized with nc82 staining (magenta). A: The entire projection pattern of the Odd neurons projecting into the calyx (∧), inferior protocerebrum (IPR) (*), and the ventromedial protocerebrum (VMPR) and posterior lateral protocerebrum (PLPR). The Odd neurons also innervate the VLPR forming a tight bundle that project toward the AMMC (white arrow). Projections are also seen crossing the midline (arrowhead). B–D: Localization of the Odd neural arbor within the different compartments of the brain (nomenclature taken from Otsuna and Ito (2006), at different anterior–posterior levels. B: At a posterior level in the brain where the Odd neurons innervate the calyx, IPR, PLPR, and VMPR. C: A section through the middle of the brain showing innervation of predominantly the IPR, the VLPR, and the anterior VMPR. D: The anterior part of the arbor of Odd projections, which are here confined to the VLPR and VMPR. E: Schematic representation of a posterior view of the Odd projection pattern. F: The entire projection pattern of the Odd neurons from a dorsal view. The cell bodies of the Odd neurons are located at the posterior aspect of the brain (arrow) and they innervate the anteriormedial protocerebrum (AMPR) (arrowhead), the IPR, and the VMPR. G: Within the dorsal aspect of the arbor the Odd projections are confined to most of the IPR region. H: Further ventrally in the brain Odd neurons also innervate the AMPR and VMPR. I: Within the ventral part of the brain the Odd neurons project into the VLPR and VMPR. J: Schematic representation of the Odd arbor viewed dorsally. SPR, superior protocerebrum; IPR, inferior protocerebrum; ca, calyx; MB, mushroom body; CC, central complex; LH, lateral horn; VMPR, ventromedial protocerebrum; VLPR, ventrolateral protocerebrum; AMMC, antennal mechanosensory and motor center; SOG, subesophageal region; AMPR, anteriormedial protocerebrum; PLPR, posterior lateral protocerebrum. Scale bar = 50 μm in A (applies to a–D) and F (applies to F–I). [Color figure can be viewed in the online issue, which is available at wileyonlinelibrary.com.]

To investigate the overall anatomy of the Odd neurons in the adult we used the Odd-Gal4 driver (Odd-Gal4 (cl)). A posterior view of the brain shows the entire distribution of the Odd arbor (Fig. 2A). The Odd neurons project into the calyx of the MB (∧ Fig. 2A) and have a large, densely packed arbor predominantly located in the IPR, PLPR (Fig. 2B), VMPR (Fig. 2B,C), and VLPR (Fig. 1C,D and * Fig. 2A). We applied the general naming system used by Otsuna and Ito (2006) for the regional subdivisions of the brain, with one exception. We use the term IPR for the entire inferior protocerebrum whereas Otsuna and Ito subdivide this area into multiple regions. The anatomical subdivisions of the brain have been schematized in Figure 2E and J. Some of the Odd neurons cross the midline (arrowhead in Fig. 2A). There is also a tight neurite bundle (arrows, Fig. 2A) projecting ventrally into the VLPR toward the AMMC. A dorsal view (Fig. 2F) shows that the cell bodies of the Odd neurons lie posteriorly in the brain adjacent to the optic lobe (arrows, Fig. 2F). The IPR projections are seen throughout the anterior–posterior (A–P) axis but appear more compact in the lateral regions (Fig. 2G,H). They also extend into the anteriormedial protocerebrum (AMPR; arrowheads, Fig. 2F). The projections into the VLPR are confined to the more medial part of this region (Fig. 2I) whereas the VMPR shows widespread Odd neural innervation although more predominantly in the dorsal aspect (Fig. 2H).

**Figure 3:**
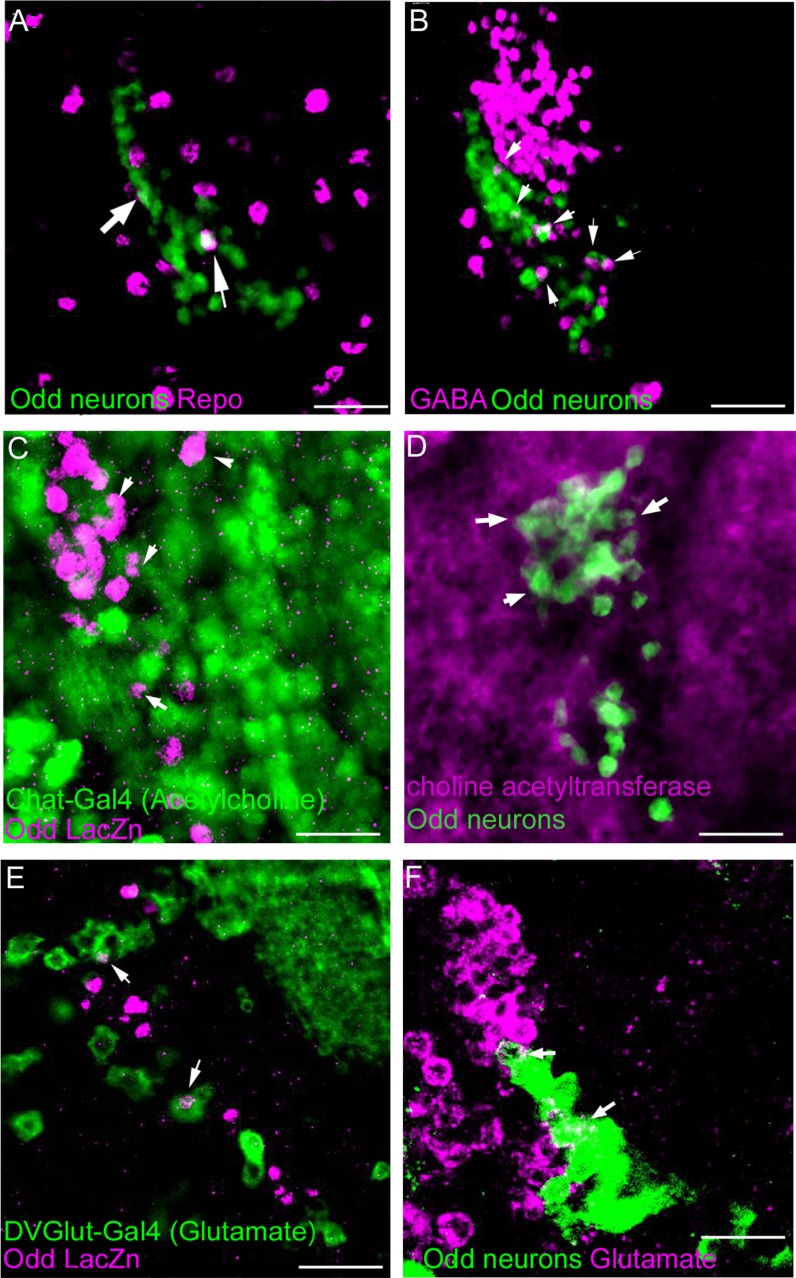
Neurotransmitter expression in Odd neurons. All images are posterior views of the brain, with lateral to the left and dorsal up. All images are ×40 magnification with a further zoom of 1.5. A: Identification of glial cells within the Odd cell population (green) using an antibody against repo (magenta). This image is a maximum intensity confocal stack composed of 10 sections, total depth of 20 μm. Only two (white arrows) of the Odd-skipped expressing cells are repo positive. B: Six of the Odd neurons (green) express GABA (magenta) as assessed by antibody staining. This image is a maximum intensity confocal stack composed of eight sections, total depth of 16 μm. C,D: Colocalization between choline acetyltransferase expressing neurons and Odd neurons, (C) using the driver line Chat-Gal4 (green) and (D) using an antibody against choline acetyl transferase (magenta). Both images are maximum intensity confocal stack composed of six sections. Both show that most of the Odd neurons are cholinergic (examples marked by white arrows), in total 68 neurons. E,F: Only two of the Odd neurons express glutamate (arrows) as assessed by (E) DVGlut-Gal4 driver (green) and (F) antibody staining (magenta). Both images are composed of eight confocal sections, total depth of 16 μm. Scale bar = 50 μm in A–F. [Color figure can be viewed in the online issue, which is available at wileyonlinelibrary.com.]

**Figure 4:**
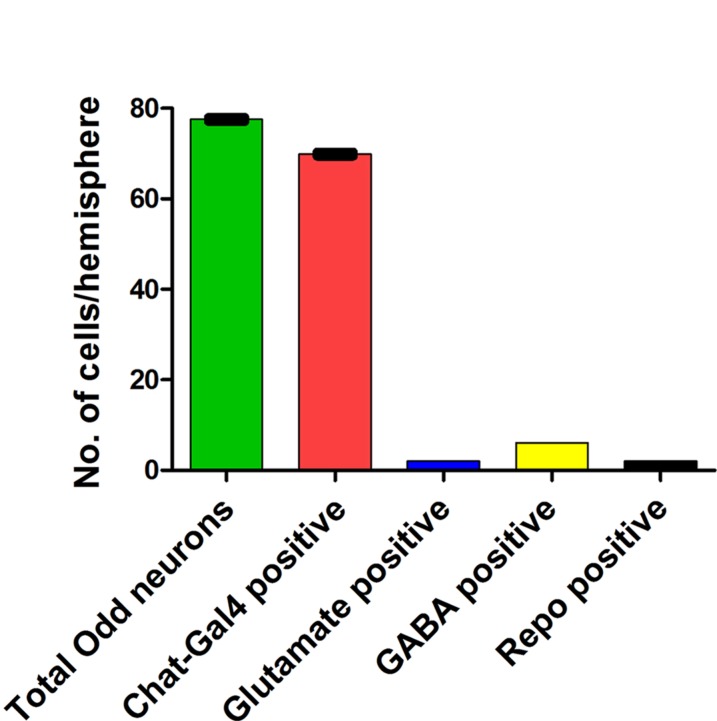
Proportion of neurotransmitter expression in the Odd neurons. Histogram showing the number of Odd neurons expressing the different types of neurotransmitters in relation to the total number of Odd neurons. *n* = 10 for each neurotransmitter. SEM was calculated for each phenotype. [Color figure can be viewed in the online issue, which is available at wileyonlinelibrary.com.]

This shows that the arbors of the Odd neurons are contained within well-defined areas of the brain. Based on the size of the arbour, we speculate that most of the Odd cells are neurons. To confirm this we assessed the number of glia in the Odd cell cluster, since glia and neurons are the only type of cells found in the *Drosophila* brain. By quantifying the number of Odd-skipped positive glia, we can deduce that the rest of the Odd cells must be neuronal. To label glia specifically we used an antibody against Reversed Polarity (Repo), a well-known glial marker (Xiong et al., 1994). Only two of the Odd cells are glia (arrows, Fig. 3A), showing that 76 of the Odd cells are neurons. To examine the type of neurotransmitters contained within the Odd neurons, we assessed the expression of the following neurotransmitters by using a combination of antibodies and Gal4 driver lines: acetylcholine, glutamate, GABA, octopamine, serotonin, dopamine, and neuropeptide F and Y. For this part of the study we took advantage of the Odd^rk111^ line, which allows us to directly compare the expression of Odd-skipped with that of Gal4 lines that label cells expressing different neurotransmitters. GABA antibody staining showed that about six of the Odd neurons are GABA positive (arrows, Fig. 3B). The most predominant neurotransmitter in the Odd neurons is acetylcholine (Fig. 3C,D). Around 90% of the Odd neurons show colocalization with the Chat-Gal4 line (arrows Fig. 3C) or an antibody that recognizes *Drosophila* choline acetyltransferase (chAT4B1) (arrows, Fig. 3D). Only two of the Odd neurons express glutamate as assessed by coexpression with DVGlut-Gal4 line (arrows, Fig. 3E), or antibody staining using an antibody that recognizes *Drosophila* glutamate (arrows, Fig. 3F). None of the other neurotransmitters are expressed in the Odd neurons. These results show that the Odd neurons are predominantly composed of excitatory cholinergic cells with a few inhibitory neurons expressing GABA (Fig. 4).

Our data show that one group of Odd neurons has distinct projections within the calyx of the MB, which is a functionally well-characterized structure of the brain. A more detailed description of this part of the arbor could provide us with information regarding the possible function of the Odd neurons in olfactory processing. In addition, a large proportion of the Odd neurons project collectively into the IPR, VMPR, VLPR, and PLPR, which are areas of the brain that are not well characterized. In our further investigations of the Odd neurons, we therefore divided the Odd neurons into two groups, those that project into the calyx and those that project into the IPR, VMPR, PLPR, and VLPR.

### One group of Odd neurons projects into the calyx and the IPR

**Figure 5:**
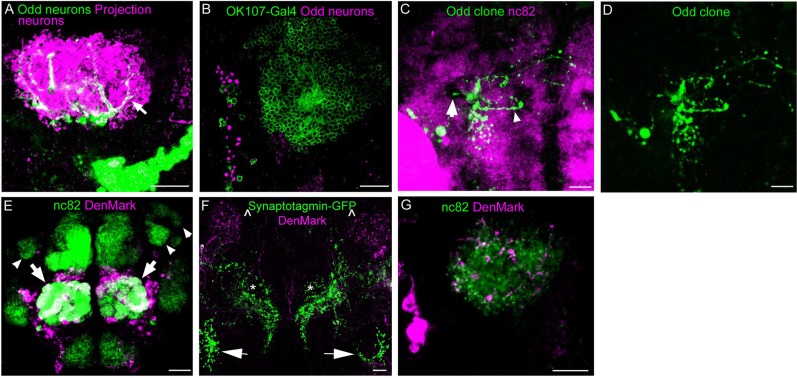
Characterization of the Odd neural projections into the calyx. All images are posterior views of the brain, expect for E, which is an anterior view, and maximum intensity confocal projections. A: Colocalization between Odd neurons (green) and projection neurons (magenta) expressing tdm-tomato in a life brain. Image is a ×40 magnification (zoom 1) composed of eight confocal sections (total depth of 16 μm). All of the Odd neurites within the calyx colocalize with the projection neurons (arrow), showing conclusively that the Odd neurons project into the calyx. B: Colocalization between Odd neurons (magenta) with the OK107-Gal4 line driving GFP (green) to label all Kenyon cells. Image is a ×40 magnification composed of 12 confocal sections (total depth of 24 μm). There is no colocalization between the Odd neurons and OK107-Gal4, showing that the Odd neurons are not part of the Kenyon cell cluster. C: Single-cell MARCM Odd clone (green) induced at 5 hours of embryonic development. Neuropil stained with nc82 (magenta). Image is ×20 magnification composed of 10 confocal sections (total depth of 20 μm). This cell projects a neurite into the calyx (arrow) and IPR. D: Single fluoroform. E: Expression of the dendritic marker DenMark in projection neurons (PNs) driven by GH146-Gal4. Image is a ×20 magnification (zoom 1.5) and is composed of a total number of 18 sections, divided into 12 anterior sections (24 μm thick) and 6 posterior sections (12 μm thick) to better visualize the calic compartment. There are approximately 30 μm between the first 12 sections and the 6 posterior sections. DenMark localizes to the dendritic portion of the PNs (arrows) as shown by DenMark immunostaining visualized with a DsRed antibody (magenta) and colocalization with antennal lobe neurons visualized with nc82 antibody staining (green). There is no antibody localization in the calyx or the lateral horn (arrowheads), indicating that Denmark localizes correctly in PNs. F: Coexpression of the axonal marker synaptotagmin-GFP and the dendritic marker DenMark in the Odd neurons specifically. Dendrites localize to the calyx (∧) whereas the IPR, VLPR, VMPR arbor is pmagentaominantly axonal (*). The projections terminating near the AMMC are also axonal (arrow). Image is a ×20 magnification composed of 15 confocal sections (total depth of 30 μm), showing that the dendritic and axonal markers localize to the different parts of the arbor. G: A high-magnification close-up (×40 magnification with a further zoom of 2) of the calyx labeled with nc82 (green) innervated by the DenMark-labeled portion (magenta) of the Odd projections. Image composed of eight confocal sections (total depth of 16 μm). Scale bar = 50 μm in A–G. [Color figure can be viewed in the online issue, which is available at wileyonlinelibrary.com.]

To describe the projection pattern of Odd neurons within the calyx in greater detail, we examined the spatial relationship between the Odd projections and the PNs that project into the calyx from the antennal lobe by using a combination of the Q and Gal4 system (Brand and Perrimon, 1993; Potter et al., 2010). This approach allows us to uniquely label the Odd neurons with GFP using our Gal4 driver, whereas the PNs are labeled separately from the Odd neurons with Tandem-dimer-Tomato using the Q system. This revealed that the Odd projections overlap with the domain occupied by PNs within the calyx (arrow Fig. 5A), and confirms that the Odd neurons project into the calyx. However, the innervation of the calyx by the Odd neurons is sparse, suggesting that only a subset of the PNs that project into the calyx could form synaptic connections with the Odd neurons. Because of the distinct innervation of the calyx, we wondered whether these Odd neurons could be a subgroup of the Kenyon cells. We addressed this by comparing the expression pattern of the Odd neurons, using the Odd^rk111^ line with that of a Gal4 lines that is known to label the Kenyon cells. We used the well-described MB-specific driver OK107-Gal4, which showed clearly that there is no overlap between the Odd neurons and the Kenyon cells (Fig. 5B). Thus the Odd neurons are not part of the Kenyon cell cluster.

We presume that the Odd neurons that project into the calyx also have projections into other parts of the brain, because most *Drosophila* neurons project more than one neurite. To visualize these projections we used the MARCM technique (Lee and Luo, 2001) to label individual cells within the Odd neural cluster. Labeling of single progeny from a cell division with GFP allows us to trace the arborizations from individual Odd neurons. As there are no anatomical landmarks to identify the Odd neurons, we chose to use the Odd-Gal driver to label clones with UAS-GFP. Therefore only Odd-skipped–expressing cells can be labeled by this approach. Gal4 expression was activated by removal of the Gal80 inhibitor during homologous recombination. Identification of multicell clones would show that the homologous recombination has occurred in the NB that gives rise to the Odd neurons. Single or double cell clones would indicate that the homologous recombination took place in the GMC. By using this approach, we were able to label three individual neurons that project into the calyx (example shown in Fig. 5C,D). These neurons project into the calyx (white arrow, Fig. 5C) and to the IPR (arrowhead, Fig. 5C). All three of the neurons project into the calyx and to different areas of the IPR. These individual neurons were labeled by heat shock induction at various points of embryonic development, whereas heat shock applied during larval stages did not produce clones of cells that project into the calyx. Thus it appears that the Odd neurons that project into the calyx and IPR are born during embryonic development. This data show that three of the Odd neurons connect the calyx with different parts of the IPR.

Next we addressed how the Odd neurons connect with the MB by establishing the axonal and dendritic proportion of the Odd neural arbor. We closely examined the different parts of the arbor to identify a dendritic or axonal specific morphology (Shepherd and Laurent, 1992). However, based on morphology alone we could not convincingly distinguish between axons and dendrites. Instead we examined the distribution of dendrites and axons among the Odd neural projections by expressing the genetically encoded dendritic marker (DenMark) with the presynaptic marker Synaptotagmin-GFP in the Odd cells using the Gal4/UAS system. The localization of DenMark to the dendritic portion of neurons has been previously described (Nicolai et al., 2010), but we sought to confirm their findings by using a driver line that labeled neurons whose polarity is well established. We chose the GH146-Gal4 line, which is expressed specifically in the olfactory PNs (Stocker et al., 1997). These neurons project dendrites into the antennal lobe and extend axons into the calyx of the MB and lateral horn. We found that DenMark exclusively localized to the dendritic portion of the PNs (Fig. 5E), which innervate the antennal lobe (arrow Fig. 5E). Furthermore, DenMark is absent from the axonal projections to the calyx and lateral horn (arrowhead Fig. 5E). This shows that in adult brain neurons DenMark localizes appropriately to dendrites. We then looked at the overall distribution of DenMark and Synaptotagmin-GFP in the Odd neurons and found that the two markers segregate within the arbor (Fig. 5F). Thus we did not observe any colocalization of the two markers within the Odd neurites, suggesting that also in these neurons the two markers are likely to localize correctly. Colocalization of the two markers could allude to one or both miss-localizing. Surprisingly, we found that DenMark localizes to the calyx of the MB (∧ Fig. 5G). Synaptotagmin-GFP is absent from this part of the arbor and so is a second axonal marker, Bruchpilot-RFP, which was tested separately (data not shown). We confirmed that the localization of DenMark is within the calyx by costaining the brains with nc82 (Fig. 5G), which labels the calyx strongly. We took advantage of the fact that DenMark is a fusion between ICAM5 and mCherry and used an antibody to DsRed to amplify the signal from DenMark (Nicolai et al., 2010) A high-resolution image (×40) shows colocalization of Denmark and nc82 staining at the calyx (Fig. 5G). Thus these data would suggest that the Odd neural projections in the calyces are dendritic.

### A second larger group of Odd neurons projects exclusively within the IPR, PLPR, VMPR, and VLPR

**Figure 6:**
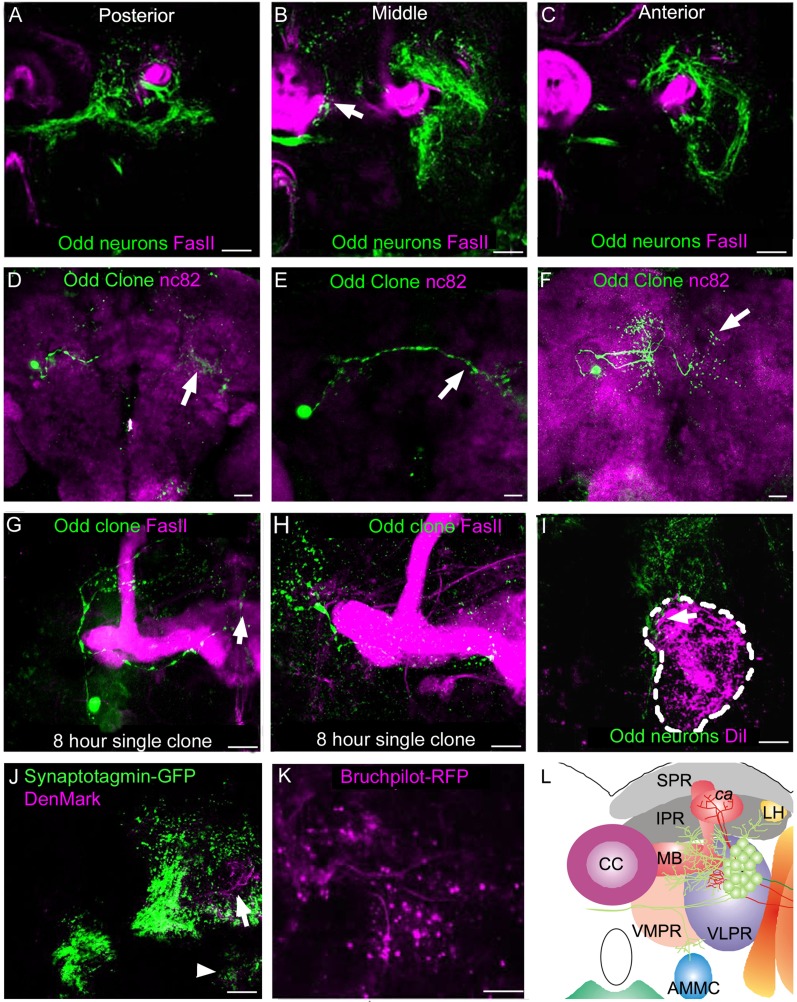
Characterization of the Odd projections into the IPR, VMPR, VLPR and PLPR. All images are posterior views of adult brains, dorsal up. A–C: Single optical sections (×40) at three different anterior–posterior levels at the level of the MB lobes. The Odd neurons appear to wrap around the MB lobes and also project toward the central complex (arrow B). D–F: Low-magnification (×20) maximum intensity stack images of single-cell MARCM Odd clones (green) in brains stained with nc82. D: Image composed of 10 sections (total depth 20 μm), showing an Odd MARCM single-cell clone induced at second instar larval stage. This neuron projects into the same compartment (VMPR) in each brain hemisphere (arrow). E: Image composed of eight sections (total depth 16 μm) showing an Odd single-cell MARCM clone induced at late second larval stage. This neuron projects into the same area within the IPR in each brain hemisphere (arrow). F: Image composed of eight sections (total depth 16 μm), showing a single-cell MARCM Odd clone induced at 7–8 hours of embryonic development. This cell projects to different areas within the same compartment in both brain hemispheres (arrow). G,H: Images (×40) of single-cell MARCM clones (green) with the neuropil stained with the FasII antibody (magenta) to label the MB. G: Image composed of eight confocal sections (total depth 16 μm). This clone was induced at first instar larval stage and shows a neuron that projects to different parts of the VMPR within the same hemisphere. H: Image composed of nine sections (total depth 18 μm). Single-cell clone that projects to the IPR and the VMPR within the same brain hemisphere. I: Life image (×40) of Odd neurons colabeled with DiI retrogradely labeled neurons (magenta) projecting into the AMMC (dashed lines). The majority of the Odd neurons lie posterior and dorsal to the AMMC, although there are a few Odd projections at the dorsal aspect of the AMMC (arrow).This image is a maximum intensity stack composed of 16 sections (total depth 32 μm). J: Coexpression of synaptotagmin-GFP and DenMark in the Odd neurons. This maximum intensity projection image (×20) is composed of six sections (total depth 12 μm). Although the majority of neurites in the IPR, VMPR, VLPR, and PLPR compartments are axonal, dendrites are also present in particular in the PLPR region (arrows). K: Another axonal marker; Bruchpilot-RFP (magenta), was used to confirm the axonal identity of the IPR, VMPR, VLPR, and PLPR arbor. This image is ×40 magnification and a maximum intensity stack composed of five sections (total depth 10 μm). L: Schematic representation of the Odd axonal (green) and dendritic (magenta) projections. SPR, superior protocerebrum; IPR, inferior protocerebrum; ca, calyx; MB, mushroom body; CC, central complex; LH, lateral horn; VMPR, ventromedial protocerebrum; VLPR, ventrolateral protocerebrum; AMMC, antennal mechanosensory and motor center. Scale bar = 50 μm in A–K. [Color figure can be viewed in the online issue, which is available at wileyonlinelibrary.com.]

A large proportion of the Odd neural arbor lies within the IPR, VMPR, PLPR, and VLPR. Little is known about these areas of the brain that border the optic lobe laterally and the central complex medially. They contain many diverse cell types, and the function of many of the neurons that project within this part of the brain is not known. To gain insight as to where the Odd neurons project within this large area we first examined their projection pattern in single confocal images at different A–P levels, focussing specifically on the area surrounding the MB (Fig. 6A–C). Odd neurons project together in bundles of fibers and appear to wrap themselves around the MB (Fig. 6A–C). Some projections also lie close to the central complex (arrow Fig. 6B). To label the MB we used FasII, which stains bundles of fibers in the brain and which labels the MB and central complex. This reveals that the Odd projections generate defined tracts and therefore that collectively the Odd neurons project to specific areas within the IPR, VMPR, PLPR, and VLPR.

To better understand the type of projections within these regions, we generated single-cell MARCM clones (Lee and Luo, 2001) at different developmental stages. This approach labeled many single Odd neurons that arborize exclusively within different compartments of the IPR, VLPR, PLPR, and VMPR (Fig. 6D–H). Again we used the Otsuna and Ito (2006) definitions of brain compartments. These clones were generated predominantly when heat shock was applied during larval stages, although a few were also labeled by inducing clones in the embryo. The types of projection patterns seen among these neurons can be broadly divided into three categories. One group of Odd neurons arborized in both brain hemispheres (Fig. 6D–F). Typically these neurons will have one small arbor in one hemisphere and another arbor located in the same compartment in the opposite hemisphere (arrows Fig. 6D–F). However, rare exceptions were noted where the arbors were morphologically different between the two brain hemispheres or innervated different compartments in each hemisphere. For example, one of the single-cell clones had a large arbor within the left hemisphere, whereas the arbor within the right hemisphere was smaller and morphologically different (arrows Fig. 6F).

A second group of Odd neurons projects exclusively within one area of the brain. An example of this is shown in Figure 6G, in which an Odd neuron arborizes exclusively within the VMPR compartment. Finally, one group of Odd neurons appears to connect different regions within the same hemisphere. For example, single Odd neurons were seen to project into the PLPR and VMPR compartments (Fig. 6H). Thus the group of Odd neurons that project exclusively within the IPR, VMPR, PLPR, and VLPR can form connections between the different hemispheres as well as between and within compartments of the same hemisphere. The projections entering the VLPR (arrow Fig. 2A) project toward the AMMC.

To assess whether Odd neurons project into the AMMC, the AMMC neurons were retrogradely labeled by injection of DiI from the Johnston organ (Lai et al., 2012). This showed that the GFP-labeled Odd neurons innervate a more medial and posterior aspect of the brain than the AMMC neurons (dashed area Fig. 6I). A small branch innervates the dorsal aspect of the AMMC (arrow Fig. 6I), but most of the Odd neurons do not innervate the AMMC, making it unlikely that they are involved in auditory processing or gravity sensing (Kamikouchi et al., 2009).

Because many of the Odd neurons project within the IPR, VMPR PLPR, and VLPR, we would expect these arbors to be a mix of dendrites and axons. This was addressed by using the dendritic marker DenMark combined with Synaptotagmin-GFP. Coexpression of the two markers shows that the IPR, VMPR, and PLPR arbors are predominantly axonal (Fig. 6J), whereas some dendritic branches could be seen intermixed throughout the VMPR and PLPR arbor (arrow Fig. 6J). We confirmed the axonal identity of these projections by using Bruchpilot-RFP (Fig. 6K), which showed a similar distribution to Synaptotagmin-GFP. The VLPR projections near the AMMC are exclusively axonal (white arrow Fig. 5F and arrowhead Fig. 6J). This data shows that dendrites are located in discrete areas within the VMPR and PLPR, whereas axons are located more broadly within the IPR, VMPR, PLPR, and VLPR (Fig. 6L).

### Three of the Odd neurons project into the lobula plate of the optic lobe

**Figure 7:**
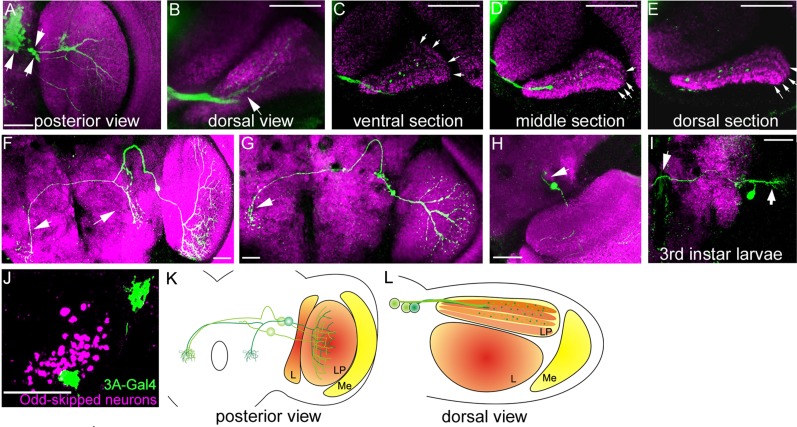
Morphology of the Odd neurons that project into the lobula plate. All images are adult brains except I. Neuropil has been stained with nc82 antibody (magenta) and the Odd neurons are labeled by GFP in green. Unless otherwise stated, all images are maximum intensity collapsed confocal z-stacks. A: A ×20 magnification image composed of 15 confocal sections (total depth 30 μm). Posterior view of the optic lobe showing the localization of the three cells (white arrows) that project into the optic lobe. B–E: Dorsal view of the lobula plate. B: Image (×40) is composed of 10 confocal sections (total depth 20 μm). The Odd neurons project into the lobula plate (LP) specifically following the outer edge of the LP. C–E: Single confocal sections through the LP showing the four layers within the LP (arrows) at three different dorsoventral positions. The Odd neurons pmagentaominantly project into the two middle layers as well as the medial aspect of the outer layer. The inner layer facing the lobula is devoid of Odd neurites. F–H: Single MARCM clones labeling the LP-projecting Odd neurons. F,G: Clones were induced early in embryonic development. F: Image is a ×20 magnification composed of eight confocal sections (total depth 16 μm). This cell projects both ipsi- and contralateral in the brain (arrows) terminating near the AMMC in both hemispheres. The projections are dense in the ventral aspect of the LP and sparser further dorsally. G: Image (×20) is composed of eight confocal sections (total depth 16 μm). This cell only projects contralateral in the brain (arrow). The projection in the LP is almost symmetrical, covering a similar area ventrally and dorsally. H: Image (×20) is composed of eight confocal images (total depth 16 μm). A third LP-projecting cell is labeled when MARCM clones are induced between 12 and 13 hours after egg lay. This cell has a similar projection pattern as G in the LP but projects a short, compact neurite into the brain. I: A ×20 magnification image composed of 10 confocal sections (total depth 20 μm). Example of the morphology of the single-cell clone shown in F and G at third instar larval stage. Already at this stage the cell appears to have adopted the morphology of the adult cell. One neurite projects across the midline (white arrow) and one toward the future optic lobe (arrowhead). J: Image is a ×40 magnification (zoom 1.5) composed of 15 confocal section (total depth 30 μm) showing that Odd neurons (magenta) do not colocalize with the 3A-Gal4 line (green), which labels HS and VS neurons. This confirms that the Odd neurons do not belong to this group of LPTCs. K,L: Schematic representation of the three LP-projecting neurons in a posterior view (K) and a dorsal view (L). LP, lobula plate; L, lobula; Me, medulla. Scale bar = 50 μm in A–J. [Color figure can be viewed in the online issue, which is available at wileyonlinelibrary.com.]

A subgroup of the Odd neurons projects into the optic lobe (arrows in Fig. 7A). By tracing the processes through consecutive confocal sections, we found that these originate from three cell bodies. Two of the cells lie close to the border with the optic lobe and the brain whereas the third is situated within the main cluster of cells. The optic lobe contains the lobula (L) and lobula plate (LP), and a dorsal view reveals that the Odd neurons specifically project into the LP (arrow Fig. 7B). To examine the localization of the Odd projections in the LP, we used the nc82 antibody to visualize the four layers in the LP (Fishbach and Dittrich, 1989). The Odd cells predominantly project into the two middle layers, whereas a few GFP-positive projections can also be seen in the medial part of the outer layer (Fig. 7C–E). The layer facing the L is devoid of Odd processes. These data show that the Odd neurons exhibit layer-specific targeting within the LP. We used MARCM lineage analysis with the aim of labeling single cells that project into the LP. We found that MARCM clonal induction at 3.5 hours after egg lay generated single-cell clones specifically labeling two of the three neurons that project into the LP. One of the LP cells projects both ipsi- and contralaterally into the VLPR and toward the AMMC in both brain hemispheres (arrows Fig. 7F). This neuron has an arbor within the ventral aspect of the LP in addition to a less dense projection into the middle and dorsal part of the LP (Fig. 6F). However, the projections do not reach the medial part of the LP.

The second of the two cells (Fig. 7G) only projects contralaterally in the brain and terminates within the VLPR near the AMMC. This neuron projects into the LP with a main branch located in the middle of the LP and what appear to be symmetrical projections extending into the dorsal and ventral aspect of the LP. The arbor of this cell seems to cover most of the LP. Thus these two neurons are morphologically distinct in terms of both the LP arbor and central brain projections. A third cell also projects into the LP with a similar type of LP arbor to that described above (Fig. 7H). However, unlike the two other cells, it has a very short compact neurite projecting into the brain terminating in the PLPR (arrow in Fig. 7H). This cell is born at a later stage in development toward the very end of embryogenesis. These data demonstrate that each of the Odd neurons that project into the LP have distinct morphologies (Fig. 7J,K).

The two contralaterally projecting Odd neurons are born as some of the first neurons in the lineage during early embryogenesis. Other types of neurons that project into the LP are born between 2 and 3 days post hatching (Scott et al., 2002), and medulla and lamina neurogenesis starts at mid-third and second instar larval stage (Huang and Kunes, 1998; Nassif et al., 2003). We therefore wondered whether the Odd neurons could play a different role in the larvae and be remodeled during metamorphosis to generate the adult projections into the LP.

To address this issue, we assessed the morphology of the single-cell clones generated from the early clonal induction at different larval stages of development. However, we found that the LP-projecting cells adopt a precise morphology from the early first instar larvae and maintain this morphology (Fig. 7I). The cell projects across the midline (as in the adult) (white arrow) and sends several thin processes toward the future optic lobe (arrowhead). Thus, these contralaterally projecting cells have a larval morphology resembling that of the adult neurons. This suggests that the function of the contralaterally projecting Odd neurons is not different in the larvae. We compared the morphology of the larval clone with that of the published map of larval brain lineages and we believe that this neuron is part of the CPI lineage (Nassif et al., 2003).

The Odd neural arbor in the LP resembles that of the HS and VS LPTCs, whereas the morphology of the projections into the central brain appears very different. HS/VS cells typically have fairly short projections into the central brain and none of them project contralaterally (Scott et al., 2002). To exclude the possibility that the Odd neurons are part of the HS/VS group of LTCPs, we compared the expression of the Odd neurons with that of a driver line that labels the HS/VS cells (3A-Gal4) (Scott et al., 2002). Again, we took advantage of the Odd^rk111^ line to label the Odd neurons separately while the HS/VS cells were labeled by GFP driven by the 3A-Gal4 line. By using this approach, we confirmed that the Odd neurons are not part of the HS/VS system of LPTCs as there is no colocalization between the two driver lines (Fig. 7J).

### The LP projections consist of dendrites and axons

**Figure 8:**
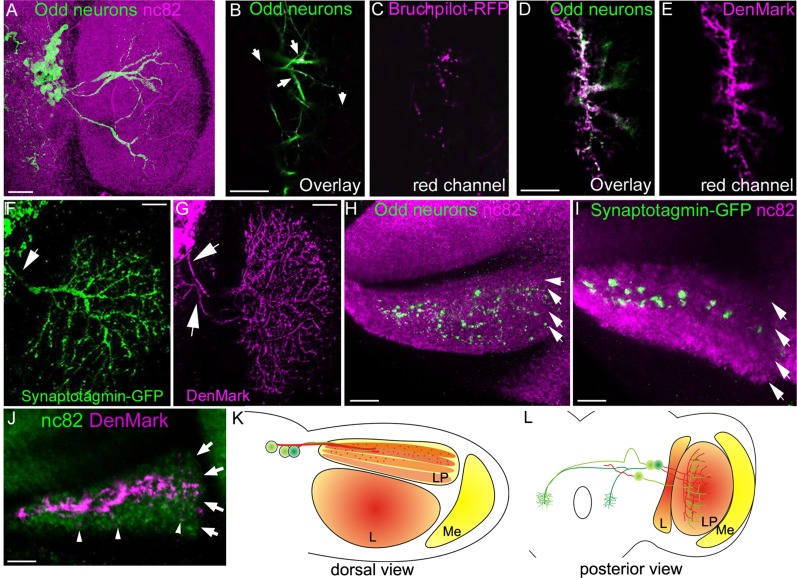
Distribution of axons and dendrites in the LP. Unless otherwise stated, all images are posterior views and confocal maximum intensity projections A: An overview of the projection pattern of the LP-projecting neurons (×40, 10 confocal sections, total depth 16 μm). The neuropil has been stained with nc82 antibody (magenta). B–E: Single optical sections through the LP. B: Expression of the axonal marker Bruchpilot-RFP (magenta) in Odd neurons labeled with GFP (green). Bruchpilot-RFP only localizes to a small fraction of the Odd projections (arrows). C: Magenta fluoroform only. D: Localization of DenMark (magenta) in Odd neurons labeled with GFP (green). The majority of the Odd arbor in the LP is dendritic. E: Magenta fluoroform only. F,G: High-magnification view (×40) of the entire LP arbor expressing either Synaptotagmin-GFP (green) (13 confocal sections, total depth 26 μm) (F) or DenMark (magenta) (13 confocal sections, total depth 16 μm) (G). F: One axonal projection can be traced from the cells (arrow). G: Two dendritic projections can be traced from the Odd neurons (arrows). H–J: Dorsal view of the LP. The neuropil has been stained with nc82 to distinguish the four layers of the LP (white arrows). All images are ×40 magnification with a further zoom of 2. H: The entire arbor of the Odd neurons labeled with GFP (green) showing the distribution of projection into the two middle layers as well as the medial aspect of the outer layer (arrows). Image composed of 20 confocal sections, a total depth of 40 μm. I: Distribution of Synaptotagmin-GFP puncta in the four layers of the LP. The axonal part of the Odd neural arbor only spans the two middle layers. Image composed of 15 confocal sections (total depth 30 μm). J: Localization of dendrites within the LP. Image composed of 15 confocal sections (total depth 30 μm). Dendrites localize to the two middle layers although some dendritic puncta can also be seen in the outer layer (arrowhead). K,L: Schematic representation of the axonal and dendritic projection of the LP-projecting Odd neurons. K: Dorsal view. L: Posterior view. LP, lobula plate; L, lobula; Me, medulla; AMMC, antennal mechanosensory and motor center. Scale bar = 50 μm in A–J. [Color figure can be viewed in the online issue, which is available at wileyonlinelibrary.com.]

To identify axonal and dendritic regions of the LP-projecting neurons (Fig. 8A), we used targeted expression of Bruchpilot-RFP, Synaptotagmin-GFP, and DenMark, respectively. First we compared the localization of each marker with that of the whole LP arbour, which was visualized with GFP. Bruchpilot-RFP localization is restricted within the LP arbor (arrows Fig. 8B,C), and many of the GFP-positive branches do not localize Bruchpilot-RFP. DenMark, in contrast, localizes to most of the LP arbor (Fig. 8D,E). This would suggest that most of the LP arbor is dendritic. As we can identify the separate primary projections into the LP, we carefully examined the entire arbor to determine how many of the cells express dendritic or axonal markers. By expressing each marker individually we could only identify one axonal projection (arrow, Fig. 8F), whereas two of the projections appear to be dendritic (arrows, Fig. 8G). Independent expression of these markers cannot reveal whether the arbors of single cells are a mix of dendrites and axons. To address this we should coexpress the two markers. However, we found that the neurons that project into the LP do so within close proximity of each other, which makes it difficult to resolve arbors from individual cells. However, our data do suggest that one cell is primarily axonal and two cells are predominantly dendritic.

In addition, we identified the localization of the axonal and dendritic branches within the LP by costaining with nc82. Axons project exclusively to the second layer and the medial part of the third layer (Fig. 8I). Thus the localization of axons is more restricted than the entire LP arbor (Fig. 8H). DenMark localization, in contrast, was seen strongly in layer 2 and 3 (Fig. 8J). Some individual DenMark puncta could also be seen in the outermost layer (arrowhead, Fig. 8J). Thus the localization of DenMark resembles that of the entire Odd arbor within the LP (compare Fig. 8H and J). This shows that dendrites are located in the second, third, and outermost layers of the LP but that the axonal input into the visual system is confined to a narrow region predominantly within the second layer (Fig. 8K,L).

### Growth of Odd neuronal projections during metamorphosis

**Figure 9:**
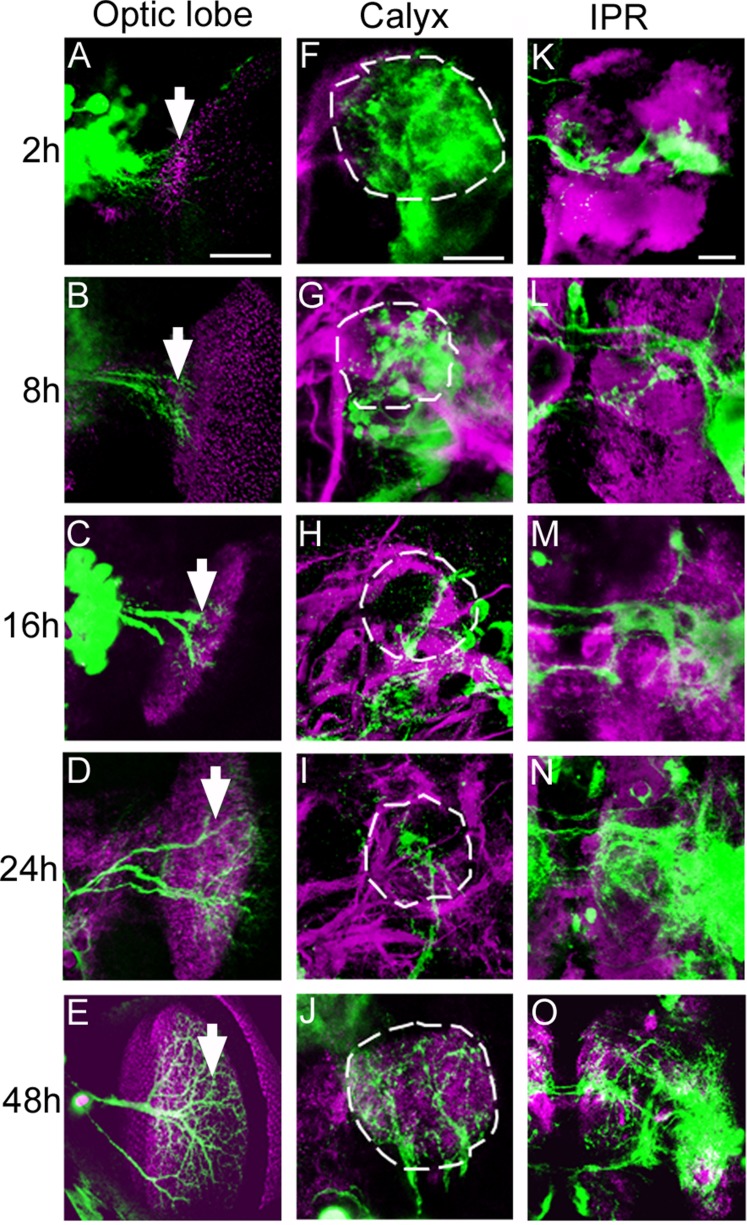
Growth and pruning of the Odd neural arbor during the first 48 hours of metamorphosis. All images are posterior views of the brain and maximum intensity projections (×40 magnification). A–E: Projections into the optic lobe. All images are composed of 10 confocal sections (total depth 20 μm). Neuropil is stained with nc82 antibody (magenta) and the Odd projections are labeled by GFP in green. A: In the first 2 hours after pupation the projections are disorganized, closely resembling that seen in the late larvae. They appear as mossy fibers. B: At 8 hours the first actual neurite can be seen (white arrow). C–E: The neurites extend while beginning to make arbors within the LP. The arbor is fully grown by 48 hours of pupation (E). The growth of the projections appears to follow closely behind the developing optic lobe. F–J: Pruning of the projections into the calyx during the first 48 hours after pupation. Brains were stained with Fas II antibody (magenta) and images are close-up views (×40 magnification) of the calyx (dashed lines). F: During the first 2 hours of pupation the Odd arbor in the calyx appear normal. G–J: Over the next 20 hours the arbor is progressively pruned away until only one branch remains (H,I). J: By 48 hours branches have been added again and branch addition is complete. K–O: Growth of the IPR, VMPR, VLPR, and PLPR arbor during the first 48 hours after pupation. Neuropil is stained with nc82 antibody (magenta). K: In the first 2 hours after onset of metamorphosis the arbor is small and compact. L–O: Over the next 46 hours the arbor grows bigger and more complex (O). Scale bar = 50 μm in A (applies to A–E), F (applies to F–J), and K (applies to K–O). [Color figure can be viewed in the online issue, which is available at wileyonlinelibrary.com.]

The projection pattern of the Odd neurons is more complex in the adult than in the larvae, where they predominantly project into the calyx (Larsen et al., 2006). Furthermore, the innervation of the calyx appears denser at larval stages than in the adult, suggesting that the Odd neurons could be pruned during metamorphosis. Pruning begins shortly after onset of pupation, and most branches have been pruned by 24 hours of pupation (Watts et al., 2004; Williams and Truman, 2005). This is followed by establishment of new connection to form the adult-specific circuits. We therefore followed the changes in Odd arborizations during pupation to address how the arbors grow and change during metamorphosis (Fig. 9). We found that the projection pattern of the Odd neurons resembled that of the adult from 48 hours after pupation and onward. The Odd neurons begin to innervate the LP as this structure begins to form (arrow, Fig. 9A–E). In the early phase, the projections appear as a bundle of small processes (Fig. 9A,B) very similar in morphology to that seen in the third instar larval brain (compare Fig. 7I with Fig. 9A).

Not until 8 hours after pupation (Fig. 9B) can we begin to see the individual branches of the LP-projecting cells. At 16 hours of pupation, only one of the three processes can be seen innervating the emerging LP (arrow Fig. 9C), but by 24 hours (Fig. 9D) all three processes have contacted the nascent LP. By 48 hours after pupation, the arbor is similar to the adult arbor (Fig. 9E). The arbor within the LP is only seen in the adult. Thus, not surprisingly, we found that these projections are not pruned. Unlike the projections into the LP, the dendrites that project into the calyx (marked by dashed lines Fig. 9F–J) of the MB are pruned during metamorphosis. Branches are pruned away (Fig. 9F–I) until only one principle branch is left at 16 hours after onset of pupation (Fig. 9H), to which new branches are added. Like the projections into the LP, maximum regrowth has taken place by 48 hours after pupation (Fig. 9J). The IPR, VMPR, VLPR, and PLPR arbors increase in complexity during the first 48 hours of pupation (Fig. 9K–O). Thus it is likely that there is a continuous addition of processes into these arbors, especially between 24 and 48 hours of pupation (Fig. 9N,O). The contralateral projections are present from the onset of pupation, and these increase in thickness during this period. Thus, unlike the projections into the calyx and to a certain extent the LP (these neurons have formed projections at larval stages), the IPR, VMPR, VLPR, and PLPR arbors are predominantly generated during pupation.

### Growth of Odd neurons during development

**Figure 10:**
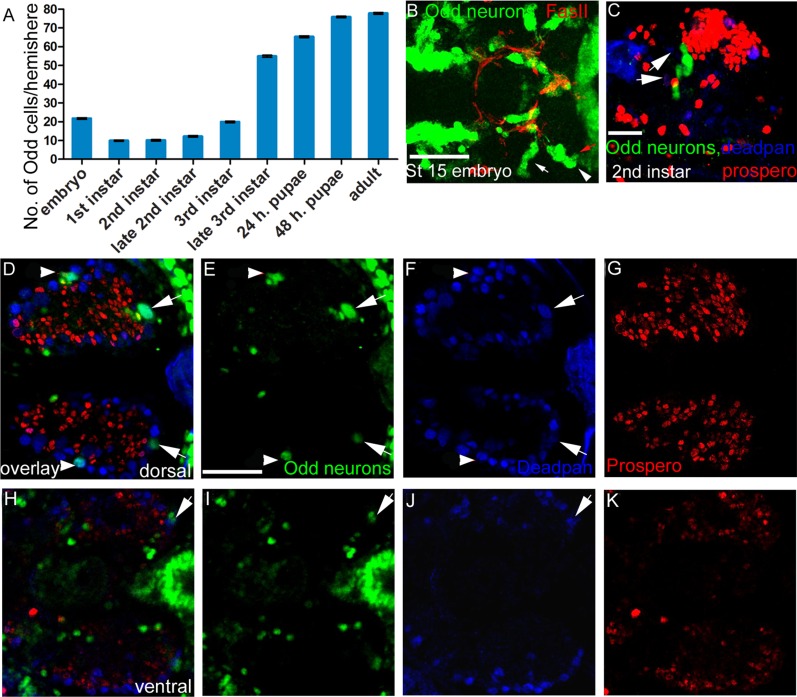
Growth of the Odd neurons. All confocal mages are maximum intensity projection. A: Histogram showing the number of Odd neurons present in the brain at different developmental stages (*n* = 10 for each developmental stage). SEM was calculated for each developmental stage. Note that the number of cells in the embryo is 22 but this number drops to 8 in the early first instar larvae. The greatest growth is seen during third instar larval stage. B: A ×40 confocal z-stack (10 sections, total depth 20 μm) image of the stage 15 embryo. Anterior is to the left. The neuropil has been stained with Fas II antibody to show the neural tracts formed in the brain. Odd-skipped (green) is expressed in four lineages: the DPL4/5 lineage (white arrow), the two MB lineages (arrowhead), and the BLP7/8 lineage (red arrow). C: Expression of Odd-skipped (green), Deadpan (blue), and Prospero (red) in the late second instar larval brain. Image is composed of six sections, (total depth 12 μm), ×40 magnification. Neither the Nb (defined by deadpan) nor the GMC (defined by nuclear Prospero) express Odd-skipped. D–K: Expression of Odd-skipped (green), Deadpan (blue) and Prospero (red) in the embryonic brain (stage 15). Anterior to the left. Each image is a single confocal section imaged at ×40 magnification. D,H: NBs (arrows) and GMC in all four lineages express Odd-skipped. D: Dorsal aspect of the brain where the DPL4/5 lineage (arrowhead) and MB1/2 lineages (white arrows) are located. H: The BLP7/8 lineage is located more ventrally (white arrow). E–G, I–K: Single fluoroform (E,I) Odd-skipped labeled by GFP (green), (F,J) deadpan (blue), and (G,K) prospero (red). Scale bar = 50 μm in B, C, and E (applies to D–K). [Color figure can be viewed in the online issue, which is available at wileyonlinelibrary.com.]

Our MARCM study revealed that the LP- projecting neurons and the neurons that project into the calyx are born during embryonic development, whereas the rest of the neurons are labeled by clonal induction at different larval stages. This would suggest that the Odd neurons are generated during both embryogenesis and larval/pupal stages. To address this issue, we first examined carefully how the cluster of cells grows by counting the number of Odd-Gal4–positive cells at different developmental stages (*n* = 10 for each developmental stage; Fig. 10A). We found the numbers to be very consistent between specimens, varying at the most by 2 cell counts. In the embryo, there are 22 cells at stage 15, but shortly after larvae have emerged, this number drops to 8. Consequently, not all embryonic Odd positive cells survive or continue to express Odd-skipped in the larvae. Most NBs go through a stage of quiescence that lasts from the end of embryogenesis through most of the first instar larval stage. This is similar for the Odd neurons, although the quiescent phase appears to last through most of second instar as well. From late second instar to crawling third instar larvae, the majority of the Odd neurons are born and about 55 of the 78 neurons have been generated by the end of the third instar larval stage. The cluster continues to grow steadily with little addition in later stages of metamorphosis.

Thus, the majority of the Odd neurons are born during later larval development and early pupae. Examination of Odd-skipped expression in the embryo (Fig. 10B) reveals that at stage 15 of embryonic development Odd-skipped is expressed in four NB lineages. We compared the expression of Odd-skipped with the embryonic lineage map (Younossi-Hartenstein et al., 2006) and found that Odd-skipped is expressed in the DPL4/5 lineage (white arrow), the two MB NBs (arrowheads), and the BLP7/8 lineage (red arrow) (Fig. 10B). These data are in agreement with those of Sprecher et al. (2007), who found a similar expression pattern of Odd-skipped using an antibody against Odd-skipped. The MB NBs generate the Kenyon cells, and we have already shown that the Odd neurons do not belong to the Kenyon cell cluster (Fig. 5B). Thus the two MB NBs that express Odd-skipped in the embryo must stop expressing the protein at some point during late embryogenesis. The number of cells in the two other lineages is exactly eight, which could indicate that it is these two lineages that generate the Odd neurons. In both these lineages (and also in MB NBs) the NBs express Odd-skipped (arrows in Fig. 10D–K). Although NBs can be identified by their larger size, we chose to define them by the presence of nuclear Deadpan and absence of nuclear Prospero, as previously described (Lin et al., 2010).

Based on these data, it is possible that the Odd neurons could be generated from both lineages or one or the other. In late second instar when the NBs have started to divide again we do not observe any Odd-skipped–positive NBs (Fig. 10C), because none of the Odd-skipped–positive neurons localize deadpan to the nucleus. Furthermore, we did not observe nuclear Prospero expression (present in the mother ganglion cells [GMCs]) in the Odd neurons at that stage, which would suggest that Odd-skipped is expressed specifically in postmitotic neurons. Thus we cannot confirm whether the Odd neurons arise from one or two NBs in the larvae.

### Odd neurons are generated as a lineage

**Figure 11:**
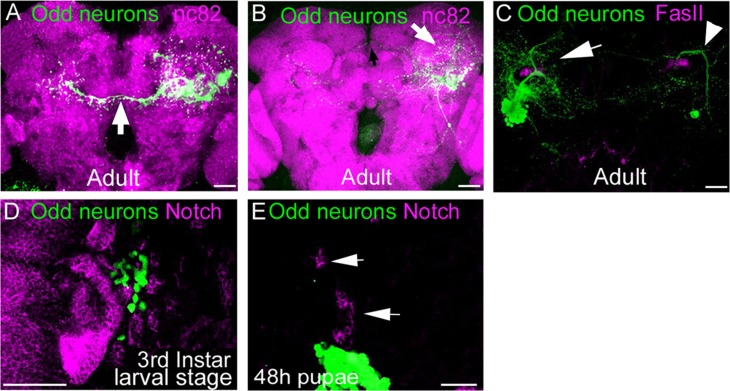
Lineage analysis of the Odd neurons. All images are posterior views of the brain and maximum intensity projections. A,B: Adult morphology of NB MARCM Odd clones induced between 3 and 4 hours of egg lay. Neuropil is stained with nc82 antibody (magenta). Both images are ×20 magnification and composed of 12 confocal sections each (total depth 24 μm). Two types of clones are generated. A: One type of clone projects across the midline just ventral to the central complex. This clone contains about 15 cells and also projects into the IPR, VMPR, and PLPR. B: The second type of clone contains about 58 cells and projects almost exclusively into the IPR, VMPR, VLPR, and PLPR within the same brain hemisphere. This clone also contains neurons that project into the calyx (white arrow). C: MARCM clone induced during first instar larval stage. Brain is stained with FasII (magenta) and imaged at ×20 magnification (14 confocal sections, total depth 28 μm).Only one type of NB clone (arrow) can be generated at this and other larval stages of clonal induction. Similarly, only one single MARCM clone (arrowhead) can be generated at each larval stage of clonal induction. D,E: Expression of Notch (magenta) in comparison with the Odd neurons (green) in the larvae (D) and 48-hour pupae brain (E). Both images are ×40 magnification; D is composed of nine confocal sections (total depth 18 μm) and E of five confocal sections (total depth 12 μm). D: The Odd neurons do not express Notch along with many other cells in the brain. E: Little Notch expression (arrow) in the 48-hour pupae brain. Scale bar = 50 μm in A–E. [Color figure can be viewed in the online issue, which is available at wileyonlinelibrary.com.]

To address whether the Odd neurons are generated from specific NBs and if so how many lineages, we generated MARCM clones to examine what type of clones were produced. When clones were induced at stage 9, we consistently labeled two types of NB clones (Fig. 11A,B). Interestingly, one type of clone (Fig. 11A) was composed of around 15 cells that send some projections across the midline (arrow) and others that wrap around the MB. The other type of clone contains 58 cells and projects almost exclusively within the same hemisphere (Fig. 11B). This type of clone consists of neurons that predominantly project into the IPR, PLPR, VLPR, and VMPR as well as projections into the calyx (arrow, Fig. 11B) and one neurite that crosses the midline in the more dorsal aspect of the IPR (black arrow, Fig. 11B). Regardless of the embryonic stage at which we induced clones, we always generated two types of NB clones. These data suggest that the Odd neurons are produced by two NBs in the embryo. However, when we induced clones at any larval stage, we only saw one type of NB clone (arrow Fig. 11C). This would suggest that at larval stages only one NB generates Odd-skipped–positive cells. Likewise, we only saw one single-cell clone at these stages (arrowhead Fig. 11C). Thus it is likely that the Odd neurons are generated from two NBs in the embryo and one in the larvae. Furthermore, the number of cells in the NB clones progressively decreased when clones were induced at later stages.

Taken together, these data strongly support the idea that the Odd neuronal cluster seen in the adult arises from defined NBs. We did not see two cell clones in any of our brains regardless of the developmental stage of clonal induction (*n* = 130), whereas we often saw single-cell clones. This strongly suggests that the Odd neurons are one hemilineage, as we would expect to see two cell clones if both GMC lineages expressed Odd-skipped. Consistent with this we see two types of single-cell clones in the embryo, one produced by each NB. In the larvae only one NB generates Odd neurons and so we only see one type of single-cell clone in each temporal window of clonal induction in the larvae.

To further confirm whether the Odd neurons are a hemilineage, we addressed whether Notch signaling is on or off in these cells. In the nerve cord and some lineages in the brain, one hemilineage has active Notch signaling whereas the other is Notch off (Lin et al., 2010), and this determines the fate of the hemilineage. Thus if the Odd neurons are indeed a hemilineage, we would expect that the cells have either Notch signaling on or off. A mixed population of Notch on and off Odd neurons would suggest that the Odd neurons are a full lineage. We began to address this question by assessing the localization of Notch in the Odd cells during larvae and pupae development using the Notch antibody that recognizes the extracellular domain (Okajima and Irvine, 2002). We chose these stages of development because the majority of the Odd neurons are born during this phase of development. However, we found that Notch is not expressed in the Odd neurons, at either early third instar larvae (Fig. 11D) or at 48 hours after pupation (Fig. 11E). In fact, many neurons in the larval and pupae brain are devoid of Notch expression. The fact that we are able to see Notch-positive cells (arrows Fig. 11E) elsewhere in the brain indicates that the antibody labeling works. Therefore we could not determine the state of Notch signaling in the brain, but we did find that all the Odd neurons were unanimous in their lack of Notch expression, confirming that the Odd neurons are a hemilineage.

## DISCUSSION

We have shown that the Odd cluster of cells contains three groups of neurons: Group one projects dendrites into the calyx of the MB and therefore likely axons into the IPR. Group two projects dendrites and axons into the optic lobe and the VLPR/PLPR. Group 3 contains a large population of neurons that exclusively project dendrites and axons within the IPR, VMPR, VLPR, and PLPR. We show that the Odd neurons are predominantly cholinergic, with a few GABAergic and glutamatergic neurons. We also provide evidence that the Odd neurons are a hemilineage generated from two NBs in the embryo but only one cell continues to produce Odd neurons in the larvae.

### Group 1: the Odd neurons that project into the calyx are likely a distinct group of extrinsic MB neurons

An interesting aspect of the Odd neurons is that a small subpopulation extends projections into the calyx of the MB and the IPR. We believe that the calic projections are dendritic based on a number of observations. First, we found that the dendritic marker DenMark specifically localizes to the calyx. Second, two independently tested axonal markers are absent from this part of the arbor. Third, the projections into the calyx are pruned during metamorphosis in a manner typical of dendrites in *Drosophila* (Williams and Truman, 2005). Collectively, these data strongly suggest that the projections into the calyx are dendritic. We acknowledge that it is possible that these markers could either miss-localize or not be expressed in the Odd neurons that project into the calyx. However, we and others (Nicolai et al., 2010) have tested the localization of DenMark and found that it is targeted correctly in neurons with known polarity. Thus we conclude that the projections into the calyx are dendritic and therefore propose that the IPR projections are axonal. Because the Odd neurons are not part of the Kenyon cell cluster, but project into the calyx, we conclude that they must be extrinsic MB neurons. Previously described extrinsic MB neurons either project dendrites into the MB lobes or send axons to the calyx (Marin et al., 2002; Pitman et al., 2011; Sejourne et al., 2011). An exception to this is MB-CP1 neuron identified by Tanaka et al. (2008), and it is possible that one of the Odd neurons that project into the calyx is MB-CP1. However, we identified up to three neurons that project into the calyx and we therefore suggest that the other two calic projecting Odd neurons are a previously not characterized group of extrinsic MB neurons.

The Odd neural dendrites innervate the dendritic portion of the MB. It is therefore conceivable that they receive synaptic input from axons that innervate the calyx. For example, they could connect with PNs carrying olfactory information to the MB from the AL. In support of this we show that the Odd neurons occupy a similar territory to the projection neurons in the calyx. Connections between Odd neurons and PNs could imply that the Odd neurons are directly involved in olfactory processing unrelated to learning and memory. Alternatively, the Odd neurons may connect to Kenyon cells via dendrodendritic synapses. In fact, presynaptic sites have been identified along the Kenyon cell dendrites (Christiansen et al., 2011). In addition, dendrodendritic synaptic connections have been found between the centrifugal horizontal (CH) and figure-ground discrimination (FD) cells in the LP in blowflies (Haag and Borst, 2002). If the Odd neurons form this type of synapse with the Kenyon cells, it is possible that the Odd neuron could function in learning and memory.

### Group 2: the LP-projecting Odd neurons are a distinct group of *Drosophila* visual neurons

Interestingly, three of the Odd neurons project into the LP. Two of these cells have large arbors in the LP and project neurites contra- and ipsilaterally into the VLPR terminating near the AMMC. A third cell also has a large arbor within the LP but projects a short neurite into the PLPR.

In flying insects, visual motion is processed by a group of cells called lobula plate tangential cells (LPTCs) (Borst et al., 2010). In *Drosophila*, the only LPTCs so far identified are the HS and VS cells that respond to wide-field motion along either the horizontal or vertical axis. Like LPTCs in other flying insects, the HS and VS cells have large distinctive dendritic arbors in the LP and project axons into multiple areas of the brain. Interestingly, the Odd neurons that project into the LP have a similar morphology to the HS and VS cells (Scott et al., 2002). However, we have shown that the Odd neurons do not belong to this group of cells. We therefore conclude that the Odd neurons are a novel group of LPTCs, because they have a similar morphology in the LP as the HS/VS cells and LTCPs identified in other dipterans (Gauck and Borst, 1999; Schnell et al., 2010; Scott et al., 2002). Our clonal analysis showed that one of the cells projects both contra- and ipsilaterally, whereas the other cell only projects contralaterally. This type of morphology would suggest that the Odd cells convey visual information not only to higher brain centers but also to both brain hemispheres.

We used the same approach to identify axonal and dendritic branches in the LP as for the calic projections. Because the arbors of individual cells lie within close proximity of each other, coexpressing the dendritic and axonal marker could not resolve whether the arbors are a mix of dendrites and axons. However, we found that most of the arbor is dendritic and were able to show that two of the projections onto the LP specifically localize DenMark. Because the contralateral projections into the VLPL are axonal, we propose that the two dendritic arbors within the LP belong to the contralaterally projecting cells.

Our clonal analysis also identified a third cell that has a short projection into the lateral brain, but a similar morphology in the LP to the other two LP-projecting Odd neurons. We believe this cell belongs to a different class of neurons, based on a number of observations. First, this neuron is born toward the end of embryogenesis, unlike the two VLPR-projecting cells that are the first neurons to be generated in the Odd lineage. It is known that in the brain distinct cell types are born at different stages of development (Kunz et al., 2012; Spletter et al., 2007; Yu et al., 2010). Thus this neuron could be specified differently from the two other Odd neurons. Second, because we believe that the two dendrites belong to the two VLPR-projecting cells, then it is feasible that the single axons we identify within the LP belong to this third cell. If this is indeed the case, then the projection from this cell into the PLPR could be dendritic. In fact, we see dendrites in this part of the brain in our dendritic/axonal coexpression studies.

Although the main dendritic projection follows the outer layer of the LP, dendrites are mostly seen in the two middle layers of the LP and the ventral aspect of the outer layer. This is unlike the VS cells that project dendrites predominantly into the outer layer of the LP (Fischbach and Dittrich, 1989). Likewise, the HS dendritic arborization is mostly seen in the LP layer closest to the lobula (Rajashekhar and Shamprasad, 2004). This further confirms that the Odd neurons are not HS and VS cells and also excludes the possibility that the Odd neurons could form direct synaptic connections with this group of cells. The axonal projections are confined mostly to the second layer of the LP. This would suggest that input from the Odd neurons into the visual system is spatially more restricted than dendritic output. The position of Odd neuron projections within the LP would allow them to form synapses with several types of neurons that project into the LP. For example, translobular-plate (Tlps) and transmedullar cells (TmYs) all display extensive arborizations within the middle layers of the LP, as well as some T cells (Fischbach and Dittrich, 1989). Thus visual information from the medulla and lobula could be sampled by the Odd neurons. Likewise, the Odd neurons could provide input to some of these groups of visual neurons

LPTCs have been described in other flying insects in terms of response properties. One such class is the figure-ground discriminating cells (FDs), which are sensitive to small moving objects but not background motion (Egelhaaf, 1985). The VLPR-projecting cells have a very similar morphology to the FD cells, especially the axonal part of the arbor. Four different types of FD cells have been identified in blowfly of which the Odd neurons resemble FD3 and FD4, respectively. It is possible that the Odd neurons could be the *Drosophila* FD-like cells. However, the LP-projecting cells could also resemble another group of blowfly LPTCs, namely, the regressive contralateral inhibited cells (rCI) (Gauck and Borst, 1999). Like the FD cells, they respond to smaller moving objects, but these cells also receive inhibitory input from the contralateral eye. Although the anatomical description of the rCI cells is not as thorough as for the FD cells, we cannot exclude the possibility that the Odd neurons could have similar properties as these.

### Group 3: the Odd neurons connect the IPR, VLPR, VMPR, and PLPR compartments

We showed using single-cell MARCM analysis that many of the Odd neurons project dendrites and axon exclusively within the IPR, VLPR, VMPR, and PLPR, although there appear to be fewer dendrites than axons. We can therefore define a subpopulation of neurons that exclusively project within this region of the brain. Our clonal analysis also showed that these neurons are predominantly born during larval and pupal stages from one NB during the period of a rapid expansion of the Odd neural lineage. It is possible that the larval NB only generates these types of neurons. In support of this we found that the Odd neural projections within the IPR, VMPI, VLPR, and PLPR progressively grow during the first 48 hours of pupation. This would suggest that the majority of the projections into these areas come from secondary neurons that are born during larval stages of development. Interestingly, many of these cells project across the midline and terminate in the same compartment in the contralateral hemisphere. It is therefore tempting to suggest that the role of these neurons is to relay information between the two brain hemispheres. Little is known about the function of this part of the brain, and this makes it difficult to speculate on the possible function of this subgroup of Odd neurons.

### The three groups of Odd neurons could be functionally related

Several studies have shown that neurons that are born from the same NB have similar functions. For example, four NBs produce the Kenyon cells, of which there are three subtypes of cells, each generated during different temporal windows of development (Kunz et al., 2012). Although the MB consists of different types of neurons, the overall function of the Kenyon cells is to consolidate memory and learning, and the subgroups of Kenyon cells are required for different aspects of memory formation. A similar situation is seen in the generation of the AL interneurons in terms of function and temporal specification (Lin et al., 2010). We demonstrate that the Odd neurons arise from two embryonic NB lineages and one in the larvae. The NBs that generate the embryonic lineages and adult lineages could in theory be different, but the projection pattern of the larval clones resembles that of clones induced in the embryo. We therefore believe that one NB stops producing Odd neurons at the end of embryogenesis, whereas the other continues through larval and early pupal development. Thus our data suggest that the Odd neurons are generated in a similar manner to other well-established neural circuits like the MB and AL interneuron lineages. These data do not provide conclusive evidence that the neurons could be functionally related but do allude to the possibility that they could.

In this respect it is interesting that not all Odd neurons express the same neurotransmitters. Although most of the neurons are cholinergic, a subset of the neurons expresses GABA, which is an inhibitory neurotransmitter. Thus there is an inhibitory component to the Odd cluster of neurons. This is the case in the AL where multiple inhibitory and excitatory interneurons shape the response properties of the projection neurons (Tootoonian and Laurent, 2010; Wilson et al., 2004).

### The Odd neurons are likely a hemilineage

The fact that we never see two cell clones (*n* = 130) strongly indicates that the Odd neurons are a hemilineage. This would also fit with the number of cells generated during larval and pupal development, which is about 60 cells. These cells are generated over a period of 4 days and considering that an average cell cycle is 1 hour (Campos-Ortega and Hartenstein, 1997), one would expect a much larger number of Odd neurons if the GMC generated two Odd neurons per division. Interestingly, in the embryo the NBs (defined by deadpan expression) express Odd-skipped, as do the GMC and progenitors. However, in the larvae neither the NB nor the GMC (Prospero positive) express Odd-skipped. This would suggest that in the larvae Odd-skipped is upregulated specifically in the postmitotic neurons. We could not establish the state of Notch signaling in the Odd neurons as they do not appear to express Notch. However, we can determine that all the Odd neurons do not express Notch and we would expect such a consistent phenotype were the Odd neurons a hemilineage.

## ROLE OF AUTHORS

All authors had full access to all the data in the study and take responsibility for the integrity of the data and the accuracy of the data analysis. Study concept and design: C.L. Acquisition of data: C.L. and P.L. Analysis and interpretation of data: C.L. Drafting of manuscript: C.L. Obtained funding: C.L.
